# Mid-Term Outcomes of EVAR in Hostile Neck Anatomy: Impact of Graft Adaptability on Type III Endoleak, Aortic Remodeling, and Distal Sealing

**DOI:** 10.3390/jcm14176226

**Published:** 2025-09-03

**Authors:** Alessandra Fittipaldi, Chiara Barillà, Narayana Pipitò, Domenico Squillaci, Giovanni De Caridi, Filippo Benedetto

**Affiliations:** Vascular Surgery Unit, Department of Medical Sciences and Morpho-Functional Imaging, University of Messina, 98100 Messina, Italy

**Keywords:** aortic remodeling, conformable endograft, distal sealing, endovascular aneurysm repair, hostile neck, type III endoleak

## Abstract

**Aim**: Hostile aortic neck anatomy—characterized by short neck length, severe angulation, conical shape, and mural thrombus or calcifications—represents a major limitation to the durability and applicability of standard endovascular aneurysm repair (EVAR). In response to these challenges, newer endografts with improved conformability have been developed. This study aimed to evaluate the mid-term outcomes of EVAR using the GORE EXCLUDER Conformable AAA Endoprosthesis (CEXC) (W.L. Gore & Associates Inc., Flagstaff, AZ, USA) in patients with hostile neck anatomy, with specific attention to type III endoleak occurrence, aortic sac remodeling, and maintenance of distal sealing. **Methods**: A retrospective observational analysis was conducted on 50 consecutive patients treated with the CEXC endograft between October 2019 and September 2023. Patients included had either elective or urgent indications for EVAR and were evaluated preoperatively using CT angiography. Hostile neck criteria were defined according to the 2019 Delphi Consensus. Procedural variables, imaging follow-up, and clinical outcomes were collected. The primary endpoints were technical and clinical success, while secondary outcomes included endoleak rates, aneurysm sac evolution, and reintervention-free survival. **Results**: Technical success was achieved in 100% of cases, with a clinical success rate of 98%. No type Ia, Ib, or III endoleaks were observed at a median follow-up of 23 months. Sac shrinkage (>5 mm reduction) occurred in 70% of patients, and distal sealing was preserved in 100% of cases. One perioperative death occurred in an emergency setting, and no late reinterventions or aneurysm-related mortalities were reported. The use of intravascular ultrasound (IVUS) and floppy guidewires contributed to precise deployment and sealing in angulated anatomies. **Conclusions**: The CEXC endograft proved to be a safe and effective option for EVAR in patients with hostile aortic anatomy, ensuring durable proximal and distal sealing, promoting favorable sac remodeling, and preventing type III endoleaks. These findings support the use of CEXC in anatomically complex settings, as long as procedures are meticulously planned and guided by appropriate intraoperative imaging and deployment techniques.

## 1. Introduction

Endovascular aneurysm repair (EVAR) has revolutionized the management of abdominal aortic aneurysms (AAAs) by providing a less invasive alternative to open surgical repair, particularly in patients with significant comorbidities. Compared to open repair, EVAR is associated with a substantially lower perioperative mortality rate, reduced hospital stay, and faster postoperative recovery, making it the preferred approach for anatomically suitable cases [1,2,3]. However, complex aortic anatomy remains a major challenge for EVAR, as up to 40–60% of patients are deemed unsuitable due to unfavorable anatomical characteristics, including short neck length, severe angulation, conical shape, and significant mural thrombus or calcifications, which collectively define a “hostile neck” according to the Delphi Consensus published in 2019 [1,4]. This anatomical complexity has historically limited the applicability of EVAR, leading to higher rates of type Ia endoleak, device migration, and the need for secondary procedures in cases with severe neck angulation and other hostile features [5]. Several contemporary EVAR platforms are specifically engineered to extend eligibility in hostile proximal necks, including Endurant II/IIs (often with EndoAnchors), Ovation iX/Alto (polymer O-rings with integral anchors), Anaconda, Aorfix (IFU up to 90° neck angulation), and Treovance (up to 75° with adequate neck length). These devices prioritize conformability, proximal fixation, and sealing in short, angulated, or conical necks, as summarized in recent technical and systematic reviews [6]. In response to these challenges, the GORE EXCLUDER Conformable Abdominal Aortic Aneurysm Endoprosthesis (CEXC) (W.L. Gore & Associates Inc., Flagstaff, AZ, USA) was developed to specifically address the limitations of conventional endografts in complex anatomies. The CEXC device is characterized by enhanced conformability, active control of proximal angulation, and repositionability, allowing for improved wall apposition even in patients with short neck lengths or proximal neck angulation up to 90° [7,8]. These features enable better adaptation to short, angulated, or irregular proximal necks. Early clinical experience and observational studies have demonstrated promising technical success rates with the CEXC in anatomically challenging cases, including patients outside the conventional instructions for use criteria.

Despite growing evidence supporting its use, under-reported yet clinically significant aspects such as type III endoleak, sac remodeling, and distal sealing remain to be thoroughly evaluated. This study aims to analyze mid-term outcomes of EVAR with the CEXC graft, focusing not only on traditional outcomes such as type Ia endoleak and mortality, but also on the stability and durability of the endograft and the maintenance of distal sealing zone integrity in patients with short and angulated aortic necks.

## 2. Methods

### 2.1. Study Design and Patient Population

This was a retrospective, single-center observational study. All patients who underwent elective or urgent endovascular aneurysm repair (EVAR) using the GORE EXCLUDER Conformable AAA Endoprosthesis (CEXC) between October 2019 and September 2023 were considered eligible. The indication for EVAR was determined through a multidisciplinary evaluation based on each patient’s comorbidities (including high surgical risk defined as American Society of Anesthesiologists [ASA] classification ≥ III), life expectancy, and individual preference.

Endograft selection was based on the CEXC’s instructions for use (IFU), which include specific anatomical criteria regarding aortic neck diameter (16–31 mm), length (≥10 mm for angulations < 60°, ≥15 mm for angulations up to 90°), and iliac anatomy (diameter 8–25 mm with a distal sealing zone ≥ 10 mm). Adequate access through iliac and femoral arteries for 12- to 18-F sheaths was also required.

The study protocol was conducted in accordance with the Declaration of Helsinki and approved by the institutional ethics committee. All subjects provided informed consent for the surgical procedure and participation in the study protocol.

Collected variables included anatomical details of aneurysm, the proximal aortic neck measurement as well as the stent graft oversize. All measurements were performed on preoperative computed tomography (CT) angiography with a maximum slice thickness of 3 mm.

The follow-up protocol included Duplex ultrasound (DUS) with contrast-enhanced ultrasound (CEUS) at 1, 3, and 6 months postoperatively, and every 6 months thereafter. All patients also underwent CT angiography at 1 and 12 months, or as indicated by the treating physician [9].

### 2.2. EVAR Procedure

Preoperative planning was conducted following the Society for Vascular Surgery (SVS) reporting standards to assess aortic pathology, and identify complex anatomical features such as neck angulation, conicity, thrombus, calcification, and iliac artery diameter and tortuosity. Key measurements also included infrarenal neck angles (β angle), as well as neck length and diameter.

Bilateral femoral artery access was achieved through surgical cutdown. Throughout the procedure, intravascular ultrasound (IVUS) (Eagle Eye Gold; Volcano Therapeutics, Rancho Cordova, CA, USA) was used to visualize the origin of the visceral vessels, assess the presence of parietal calcification and thrombus, and optimize diameter and length measurements [10].

To ensure optimal conformity of the endograft to the aortic curvature and to achieve precise alignment with the aortic neck, the use of floppy guidewires may be employed. Alternatively, a stiff guidewire can be used and partially withdrawn at the time of deployment, so that the floppy tip corresponds to the aortic neck during release. These tools facilitate smoother navigation through tortuous anatomies and reduce the risk of malposition, especially in cases of severe neck angulation. Their flexibility supports proper endograft orientation and deployment, complementing the CEXC device’s active angulation control capabilities designed to enhance conformability in hostile proximal necks. As commonly observed in patients with complex neck anatomy, a slight distal migration of the stent graft often occurred during deployment. In such cases, the device was re-constrained and straightened, allowing for accurate repositioning and redeployment just below the lowest renal artery. An intermediate angiographic assessment was routinely performed at this stage to confirm the proper alignment of the endograft in relation to the renal artery. Following the deployment of the contralateral limb, the active angulation control was released, and the delivery system for the main body was withdrawn. A final angiographic evaluation was then carried out to verify optimal graft positioning, ensure patency, and exclude the presence of kinking or endoleaks.

### 2.3. Statistical Methods and Outcomes

Analyzed outcomes included perioperative and follow-up complications. Early outcomes included the use of intraoperative unplanned cuff, technical success, 30 days mortality and morbidity. Late outcome included Type I/II/III endoleaks, aortic neck dilatation, aneurismal sac shrinkage and reinterventions.

Continuous variables were reported as or median with interquartile range (Q1, Q3). Categorical variables were reported as count (percentage). Statistical analysis was performed using R, version 3.3.3 (R Core Team, 2017) (The R Foundation for Statistical Computing, Vienna, Austria; https://www.r-project.org/ accessed on 15 March 2024).

## 3. Results

Between October 2019 and September 2023, a total of 50 patients underwent EVAR using the GORE EXCLUDER Conformable AAA Endoprosthesis (CEXC) at our Institution. All procedures were performed with a mobile C-arm. Table 1 and Table 2 provide a summary of patients’ risk factors and the anatomical characteristics of the treated abdominal aortic aneurysms. The median age at the time of the procedure was 72.1 years (Q1, Q3 [67.1, 80]), and 90% of patients were male. In our patient cohort, 10 (20%) patients met all the criteria for a hostile aortic neck as defined by the 2019 Delphi Consensus, while 28 (56%) patients presented with at least one of these features. The criteria include short neck length (<15 mm), severe angulation (>60°), conical shape, large diameter (>28 mm), and the presence of significant mural thrombus or calcifications.

All but three procedures were performed electively. The median operative time was 110 min (Q1, Q3 [100, 145]), with a median fluoroscopy time of 21 min (Q1, Q3 [17, 33]). In three cases, an iliac branch device was used. A 0.035-inch floppy guidewire (Runthrough NS Floppy, Terumo, Tokyo, Japan) was used in 14 cases (28%). IVUS was used in 7 (14%) of cases to evaluate seal zone adequacy and perform accurate diameter and length measurements in more complex aortic neck anatomies. Intraoperative AAA-sac coil embolization was performed in 15 (30%) of cases in patients with high risk for type II endoleak (absence of circumferential sac thrombus, two or more pairs of patent lumbar arteries, patent inferior mesenteric artery) [11,12].

Table 3 provides a summary of primary and secondary endpoints. Technical success, defined as successful stent graft deployment without type I or III endoleak at the completion angiography, was achieved in 100% of cases. Clinical success—defined as technical success without intraoperative conversion or mortality—was achieved in 49 (98%) of cases. The only exception was a patient who underwent emergency repair for a ruptured AAA but died intraoperatively from hemorrhagic shock.

Within 30 days post-procedure, only one patient experienced a decline in renal function. DUS and CEUS raised suspicion of malposition of the main body of the stent graft and partial occlusion of the left renal artery, which was subsequently confirmed by CT angiography. The patient was successfully treated with percutaneous transluminal angioplasty and stenting, resulting in improved renal function. No early mortality (within 30 days) was detected.

At a median follow-up of 23 months (Q1, Q3 [18, 38]), no type Ia, Ib or III endoleaks occurred. Late persistent type II endoleak was recorded in 10 patients, of whom 3 showed sac shrinkage and 7 demonstrated sac stability. None required surgical intervention.

The death rate during follow-up was 4 patients (8%), with no aneurysm-related fatalities.

Aneurysm sac shrinkage (defined as a reduction in sac diameter > 5 mm) was documented in 35 (70%) of patients, while stabilization was noted in 15 (30%).

Distal sealing, defined as the absence of type Ib endoleak and stable iliac seal, was maintained in 100% of patients. No late reinterventions were necessary.

Overall survival, estimated by Kaplan–Meier analysis, was 90% at 12 months and 87% at 24 months following EVAR with CEXC. Freedom from type II endoleak was 89% at 12 months and decreased to 76% at 24 months, with most events occurring during the first two years of follow-up.

**Figure 1:**
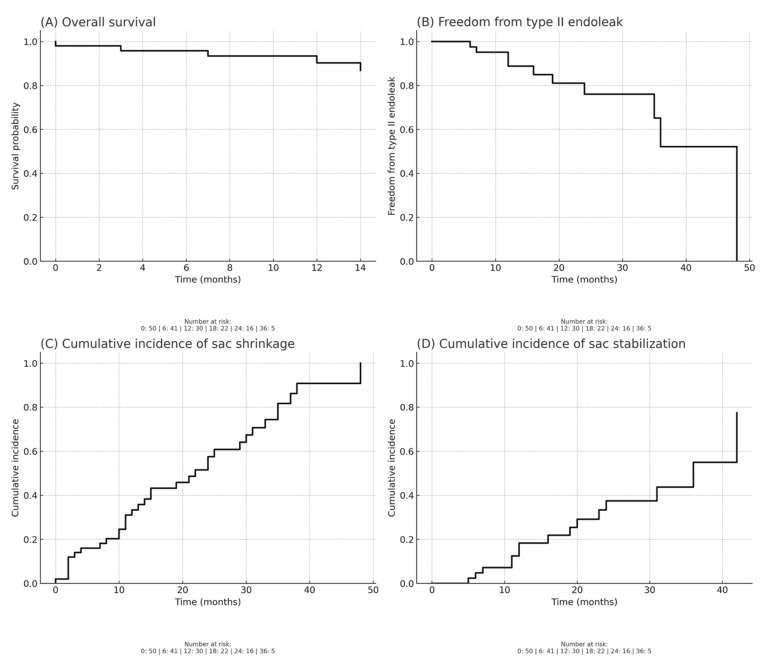
Kaplan–Meier life-table analysis. (**A**) Overall survival. (**B**) Freedom from type II endoleak. (**C**) Cumulative incidence of sac shrinkage. (**D**) Cumulative incidence of sac stabilization.

Regarding sac remodeling, the cumulative incidence of sac shrinkage was 33% at 12 months and increased to 58% at 24 months, while the cumulative incidence of sac stabilization reached 18% at 12 months and 37% at 24 months. Figure 1 shows Kaplan–Meier analysis.

## 4. Discussion

The GORE EXCLUDER Conformable AAA Endoprosthesis (CEXC) has emerged as a significant innovation in endovascular aneurysm repair, offering viable solutions in anatomically challenging scenarios where standard endografts often fail. The existing literature has predominantly focused primarily on proximal neck stability and type Ia endoleaks. Our analysis emphasizes the importance of distal sealing and type III endoleak prevention, which remain underreported yet critical for long-term device durability.

Short necks (<15 mm) and severe angulations (>60°), long regarded as exclusion criteria for conventional endografts, are increasingly manageable with CEXC [13,14]. The Triveneto Conformable Registry reported a 100% technical success rate in patients with severe neck angulations (mean 68.9°), with no type Ia endoleaks and no device-related complications at 30 days [15]. Our findings align closely with the preliminary experience described by Bonvini et al., who implemented a standardized through-and-through axillo-femoral approach and selective renal artery cannulation to achieve optimal deployment in cases with angulations up to 122°. In our patient cohort, we used the Terumo floppy guidewire (Runthrough NS Floppy, Terumo, Tokyo, Japan) in patients with an aortic angulation ≥ 60° to facilitate adaptation to the aortic curvature and achieve precise alignment with the aortic neck and IVUS (IVUS, Eagle Eye Gold; Volcano Therapeutics, Rancho Cordova, CA, USA) to optimize the selection of the proximal sealing zone.

In hostile-neck EVAR, evidence reports type Ia endoleak rates of ~2.65% at 30 days and ~6.65% at mid-term, with mean migration ~1.86%, despite frequent outside-IFU use.

On this basis, platforms tailored for hostile necks show distinct performance profiles: Aorfix reached 96.3% technical success with rising reinterventions to 17% at 5 years and few early type I/III endoleaks; Anaconda reported 98.3% technical success with 5.7% type I and 22.7% type II endoleaks at 60 months; Endurant II/IIs achieved 99.3% technical success with 0.8% type I, 0% type III, and 11% reinterventions at 60 months; Ovation showed 99.7–100% technical success but 4.9–6% type I/III endoleaks and 15–15.5% sac enlargement at 5 years, whereas Alto reported 1.3% type Ia at 12 months; and Treovance demonstrated ~96% clinical success with low early reintervention [6,16].

Early CEXC series similarly show 100% technical success, no type I/III endoleaks at short-term, and a predominance of type II endoleaks [15].

In our cohort treated with CEXC, we observed 100% technical success, 0% type I/III endoleaks at a median 23 months, 70% sac shrinkage, no reinterventions, and 100% distal sealing—comparing favorably with these benchmarks while acknowledging differences in design, indications, and surveillance.

While IFU guidelines provide a safe operational range (neck angulation ≤ 60° with neck length ≥ 10 mm, or ≤90° with ≥15 mm neck) [14], clinical experience demonstrates that carefully executed deviations can broaden CEXC’s utility. Vacirca et al. [17] showed provided procedural adaptations, such as pre-deployment ballooning and angulation control, which enhance sealing in highly tortuous anatomies. However, they also emphasize that improper support or poor wire selection can lead to deployment inaccuracies, underscoring the importance of technique refinement. In our patient cohort, all procedures were performed in accordance with the IFU for CEXC [14].

The EXCeL Registry [18] demonstrated the effective proximal apposition of the CEXC, but did not fully address the challenges associated with distal sealing in highly angulated or short neck anatomies. Our data suggest that achieving and maintaining adequate distal sealing is crucial for preventing late failures, especially in patients with complex aortic anatomies. In our cohort of 50 patients treated with the GORE EXCLUDER Conformable AAA Endoprosthesis, we observed no type Ib endoleaks or iliac-related reinterventions during a median follow-up of 23 months. This is a notable outcome, especially considering that 56% of patients presented with at least one hostile aortic neck characteristic, and 20% fulfilled all criteria for a hostile neck as per the 2019 Delphi Consensus. Despite the anatomical challenges, distal sealing remained durable in all cases, underscoring the importance of careful preoperative planning and intraoperative techniques.

Importantly, survival and complication rates in our series confirm the long-term safety profile of the device. Kaplan–Meier estimates demonstrated overall survival of 90% at 12 months and 87% at 24 months, while freedom from type II endoleak was 89% at 12 months and decreased to 76% at 24 months, with most events occurring in the first two years. Regarding sac remodeling, the cumulative incidence of sac shrinkage was 33% at 12 months and increased to 58% at 24 months, whereas the cumulative incidence of sac stabilization reached 18% at 12 months and 37% at 24 months. These findings provide further evidence of the device’s capacity to promote durable sac remodeling and long-term anatomical stability. Consistent with this, we observed aortic remodeling—defined as notable sac shrinkage in the absence of new endoleak formation—in 27 (54%) of patients, an important surrogate marker of long-term procedural success. The consistent performance of both the aortic remodeling and the distal sealing may be attributed to multiple technical strategies, including appropriate sizing, the use of iliac branch devices when necessary, and, in selected cases, IVUS to optimize assessment of the landing zones and the use of guidewire techniques to enhance device conformity and positioning. These findings are consistent with the EXCeL registry [18], which reported sac regression in 55.1% of patients at 12 months post-procedure, with no new type I or III endoleaks. Similarly, Bonvini et al. [14] further confirmed favorable early remodeling outcomes, reporting no sac enlargement or type Ia endoleaks in patients with severe neck angulation, further confirming the capacity of the CEXC device to promote favorable remodeling even in anatomically challenging cases.

Our results add to the increasing evidence that while proximal sealing often receives more attention due to its association with type Ia endoleaks, distal sealing should not be underestimated, especially in patients with tortuous or ectatic iliac arteries. Durable distal sealing is essential not only for preventing type Ib endoleaks but also for avoiding migration and preserving long-term clinical success without the need for secondary interventions.

Type III endoleak were also investigated, as they represent a less frequent but potentially serious complication, with a substantial risk of aneurysm rupture if not promptly addressed. The use of flexible guidewires and active angulation control, as described by Hiroyuki Nakayama et al. [19], may be particularly beneficial in this context, as it can reduce the risk of proximal malapposition and subsequent type III endoleak formation. In our experience, the strategic use of a 0.035-inch floppy guidewire—or alternatively, the partial withdrawal of a stiff guidewire to expose its floppy tip during deployment—proved effective in enhancing graft conformability and ensuring proper apposition to the aortic wall at the proximal neck. This technique facilitates a more controlled alignment of the endograft with the native aortic curvature, particularly in angulated or hostile necks, thereby minimizing the potential for incomplete sealing or component separation.

Given the anatomical variability in different patient populations, this study provides important real-world evidence that can guide device selection and procedural planning in similar patient groups.

## 5. Conclusions

This study reinforces the GORE EXCLUDER Conformable Endoprosthesis (CEXC) as a reliable and versatile solution for EVAR in patients with hostile aortic anatomies. Despite encouraging mid-term outcomes—marked by high technical success, effective proximal sealing, and significant aortic remodeling—ongoing surveillance remains critical to identify and manage late complications such as type II endoleaks. While off-label applications beyond IFU appear technically feasible, their safe adoption depends on operator experience, procedural standardization, and structured follow-up. Continued multicenter studies and long-term data are needed to further validate these approaches and optimize outcomes in complex anatomical settings.

## 6. Strengths and Limitations

This study has several strengths, including the standardized procedural planning, and systematic imaging follow-up, which strengthen the reliability of the reported outcomes. However, some limitations should be acknowledged. The retrospective, single-center design may introduce selection bias and limit generalizability. Additionally, the relatively small sample size (*n* = 50) reduces the statistical power to detect less frequent adverse events. Finally, the median follow-up of 23 months, while sufficient to assess mid-term outcomes, may not capture very late complications or long-term durability of the device. Despite these limitations, our findings provide valuable real-world evidence supporting the role of the GORE EXCLUDER Conformable Endoprosthesis in anatomically challenging cases and add meaningfully to the growing body of knowledge in this field.

## Figures and Tables

**Table 1:** Demographics and risk factors of the 50 patients treated with CEXC for AAA. chronic obstructive pulmonary disease (COPD); transient ischemic attack (TIA). Values are *n* (%) or median (interquartile range).

Demographics	
Age	72.1 (Q1, Q3 [67.1, 80])
Male	45 (90%)
**Risk Factors**	
Hyperlipidemia	39 (78%)
Diabetes	5 (10%)
Hypertension	39 (78%)
End stage renal disease	12 (24%)
COPD	10 (20%)
Previous Myocardial Infarction	14 (28%)
Previous TIA/stroke	2 (4%)

**Table 2:** Anatomical characteristics and intraprocedural data of the 50 patients treated for AAA. Values are *n* (%).

Aortic Proximal Neck	
Length	15 mm (Q1, Q3 [8, 25 mm])
Diameter (max)	24 mm (Q1, Q3 [19, 31 mm])
Beta-angle	55° (Q1, Q3 [20°, 90°])
Conical shape	21 (42%)
>50% calcification	19 (38%)
>50% thrombus	13 (26%)
**Intraprocedural data**	
Proximal diameter main body	29 mm
Oversize%	18% (Q1, Q3 [14, 21])
Repositioning system	16 (32%)
Angulation control system	33 (66%)
Floppy guidewire	15 (30%)
Floppy tip of stiff guidewire	7 (14%)
Coils embolization	9 (18%)
Iliac branch	3 (6%)
Proximal cuff	0

**Table 3:** Primary and secondary endpoints. Median follow up was 23 months (Q1, Q3 [18, 38]). Values are *n* (%).

Primary Endpoints	
Technical success	50 (100%)
Clinical success	49 (98%)
**Secondary endpoints**	
Endoleaks	
Type I a/b	0
Type II	10 (20%)
Sac shrinkage	3 (30%)
Sac stability	7 (70%)
Type III	0
Aneurysm sac shrinkage	35 (70%)
Aneurysm sac stability	15 (30%)
Death rate	4 (8%)

## Data Availability

The data that support the findings of this study are available from the corresponding author, A.F., upon request, due to privacy reasons.
